# An Innovative Course on Involving Patients in Health Professions Education

**DOI:** 10.5334/pme.1190

**Published:** 2024-08-02

**Authors:** Kaylee Eady, Catherine Giroux, Sarah Heath, Katherine A. Moreau

**Affiliations:** 1 Faculty of Education, University of Ottawa, Ottawa, ON, CA; 2 Department of Criminal Justice, University of Winnipeg, Winnipeg, MB, CA

## Abstract

**Background & Need for Innovation:**

Patients can be actively involved in various aspects of health professions education (HPE). However, learners in HPE graduate programs have minimal opportunities to learn how to involve patients in HPE.

**Steps Taken for Development and Implementation of Innovation:**

We designed, implemented, and evaluated a 12-week asynchronous, online graduate course that provides learners such opportunities. We established an advisory committee of patients, clinician-educators, and professors to guide course development. Using Thomas et al.’s framework, we established the general and targeted need for the course, identified the learning outcomes, determined the learning activities, and implemented and evaluated the course. It is offered within the asynchronous, online Diploma and Master in HPE at the University of Ottawa, Canada.

**Evaluation of Innovation:**

Forty learners participated in the course between 2020 and 2022. Using a survey with closed- and open-ended items, learners reported satisfaction with all course components, and they valued the patient narrative videos created for the course. After course completion, learners reported that the course is relevant to their professional practice. They also reported confidence in their abilities to actively involve patients in HPE. Based on the culminating assignment assessment data, learners attained course expectations.

**Critical Reflection:**

Although patients who participated in the narrative videos represented diverse age ranges, health conditions, and experiences in HPE, they were often Caucasian, educated, and from a higher socio-economic background. Also, the level of engagement between patients and learners in the course was limited. We are committed to improving our own patient involvement efforts.

## Background and Need for Innovation

Patients and family members (herein referred to as patients) can actively contribute to health professions education (HPE) by, for example, teaching and assessing health professionals and health professions learners, assisting with curricula development, and participating on student admissions or faculty hiring committees [1]. Active patient involvement in HPE has direct benefits to patient care, including enhanced patient-clinician communication, enriched clinical reasoning, and improved shared decision-making [2,3]. Similarly, practicing patient- and family-centred care increases compassion and humanism in healthcare. To facilitate learners’ acquisition of the attitudes and behaviors needed to practice patient- and family-centred care, it is necessary to model these by actively involving patients in processes for health professions learning [3].

Yet, patient involvement in HPE has been sporadic and inadequate, prompting Towle et al. [4] to recommend that educational institutions prioritize patient involvement, promote sustained involvement opportunities for patients, and increase the diversity of those advocating for patient involvement. Rowland et al. [5] caution the problematic state of the literature on patient involvement and further note that it risks limiting the conceptualization of patient involvement to *patients as teachers*. They conclude by advocating for careful and collaborative thought to advance understanding and practices. We believe that another contributing factor is that learners in HPE graduate programs have minimal opportunities to engage in learning on patient involvement in HPE, and in reflection on their patient involvement practices or aspirations as current or future clinician-educators [1].

### Goal of Innovation

To address this problem, we designed, implemented, and evaluated a 12-week asynchronous, online graduate course that provides learners with information on and strategies for actively involving patients in HPE. It is tailored to learners enrolled in the asynchronous, online Diploma or Master in HPE at the University of Ottawa, Canada.

## Steps Taken for Development and Implementation of Innovation

We established an advisory committee consisting of two of each patients, clinician-educators, and university professors to guide course development and content. We used Thomas and colleagues’ [6] curriculum development approach to inform our process. Specifically, we: (1) conducted a general needs assessment, (2) conducted a targeted needs assessment, (3) identified the learning outcomes, (4) established educational strategies to achieve the learning outcomes, (5) implemented the course, and (6) evaluated the course. The University of Ottawa’s Office of Research Ethics and Integrity deemed the course development, its evaluation, and dissemination as quality improvement and thus, it was exempted from ethical review.

### General and Targeted Needs Assessment

We conducted a general needs assessment that gathered, reviewed, and synthesized material related to strategies for actively involving patients: (a) in HPE and (b) in the education of clinician-educators on patient involvement in HPE. Subsequently, we conducted a targeted needs assessment that explored the interests of learners enrolled in HPE graduate programs at our institution in learning how to involve patients in various aspects of HPE. We also explored their perceptions of the importance of involving patients in HPE and the challenges associated. Finally, the targeted needs assessment helped elucidate learners’ previous learning on how to involve patients in the education of health professionals, if any at all. The findings of these needs assessments, which are not reported herein, informed the development of the learning outcomes, as well as the educational strategies for the course.

### Course Learning Outcomes and Educational Strategies

Upon completion of the course, learners should be able to: (1) critically reflect on theory and current evidence on active patient involvement in HPE; (2) teach others about active patient involvement in HPE; (3) create innovations that actively involve patients in HPE; and (4) evaluate innovations that actively involve patients in HPE.

The course consists of 12 weekly asynchronous, online modules, each focusing on one of 12 essential questions (See Table 1) intended to trigger learners’ reflection on the active involvement of patients in HPE. Required readings, consisting of published peer-reviewed literature relevant to the given essential question, form the foundation of each weekly module. Learners then engage with a patient narrative video, a unique and defining feature of each module, in which patients provide their answers to and reflections on the given essential question and share their personal experiences of being involved in HPE. To develop these videos, we recruited 28 adult patient advisors from a provincial patient advisory program focused on patient engagement in healthcare. Any patient who was part of this program was eligible to participate. We edited each of the 12 videos, which are approximately 20-minutes long, to present a compilation of multiple patients’ answers, reflections, and experiences. Importantly, learners can connect with the patients, upon request and with the professor’s facilitation, if they wish to learn more about their experiences. Following the patient narrative video, learners complete a learning activity to apply the concepts related to the weekly module. Each activity is unique to the module, involves learners in both individual and peer-peer learning, and engages them in reflection, brainstorming, critiquing, idea sharing, discussion, analysis, and/or application to their contexts. We provide them with the objective, instructions, necessary resources (e.g., report or policy to critique), and space to engage with peers (e.g., online discussion boards, chat rooms).

**Table 1:** Learners’ Perceived Relevance to Professional Practice.


ESSENTIAL QUESTION	N	NOT AT ALL RELEVANT	RELEVANT TO A SMALL EXTENT	RELEVANT TO A MODERATE EXTENT	RELEVANT TO A GREAT EXTENT	I DON’T KNOW

		n (%)				

What does patient involvement in HPE mean to you?	20	0	2 (10)	5 (25)	13 (65)	0

Why should we involve patients in HPE?	20	0	1 (5)	4 (20)	15 (75)	0

How can we involve patients in HPE?	20	0	1 (5)	7 (35)	12 (60)	0

How can we involve patients in the development of admissions criteria/processes as well as the development of educational curricula?	20	1 (5)	7 (35)	7 (35)	4 (20)	1 (5)

How can we involve patients in teaching and mentoring activities in HPE?	20	2 (10)	1 (5)	7 (35)	10 (50)	0

How can we involve patients in assessment and the provision of feedback in HPE?	20	1 (5)	2 (10)	7 (35)	10 (50)	0

How can we involve patients in HPE research?	20	2 (10)	3 (15)	6 (30)	8 (40)	1 (5)

What do learners and clinician-educators think about patient involvement in HPE?	20	0	2 (10)	6 (30)	12 (60)	0

What do patients think about patient involvement in HPE?	20	1 (5)	0	6 (30)	13 (65)	0

What are the consequences (positive and negative) of patient involvement in HPE?	20	0	1 (5)	3 (15)	16 (80)	0

What innovations can facilitate patient Involvement in HPE?	20	0	1 (5)	8 (40)	11 (55)	0

How can we evaluate innovations that facilitate patient Involvement in HPE?	20	0	1 (5)	8 (40)	11 (55)	0


Throughout the course, the learners work individually or in pairs to create an innovation (e.g., an activity, a program, a process), as a culminating assignment, that actively involves patients in HPE. While it is not mandatory that they implement this innovation in practice, we encourage them to do so and support them in ensuring that their innovation is something that they or someone else could feasibly implement in a HPE context. They prepare an innovation plan and a presentation that their peers and the professor assess. In their plan they address: (a) What is the innovation? (b) What problem/issue does the innovation address? (c) How will the innovation actively involve patients in HPE? (d) Why is the innovation needed and important? (e) What are the potential consequences (positive and negative) of the innovation? (f) How will the innovation be evaluated?

### Course Implementation

The course is an elective offering within our institution’s asynchronous, online Diploma and Master in HPE and thus, we deliver the course asynchronously using our institution’s course learning management system. The course is offered over 12 consecutive weeks, with each week covering one module. We implemented the course for the first time in the Winter 2020 term and have offered it every subsequent year. It started as a Special Topics course and has now become a permanent offering in our institution’s HPE graduate programs.

## Evaluation of Innovation

The major evaluation questions for the course include: (1) Are learners satisfied with the course? (2) Following course completion, to what extent do learners perceive the course to be relevant to their professional practice? (3) Following course completion, to what extent are learners confident in their abilities to actively involve patients in HPE? (4) How did learners perform on the culminating assignment? We surveyed learners at the end of each course offering to answer questions 1 to 3 and reviewed assessment data for the culminating assignment to answer question 4. Participation in the evaluation was voluntary. We conducted a conventional content analysis on all open-ended survey responses to identify key comments related to the patient narrative videos [7]. We also conducted descriptive statistics on all closed-ended survey items and on assessment data for the culminating assignment. As our institution’s graduate HPE programs continue to grow and enrollment in this course steadily rises, we will use these factors and the findings of the ongoing evaluation to engage in a process of continuous quality improvement. The findings from 2020–2022 serve as the basis of the findings reported herein.

### Findings

Between 2020–2022, 40 learners participated in the course. Of these learners, 20 completed the post-course evaluation survey. Of those that completed the post-course evaluation survey, 3 (15%) had been involved in teaching, supervising, and/or assessing learners in HPE for less than 1 year, 9 (45%) had been involved for 1–5 years, 3 (15%) for 6–10 years, 4 (20%) for 11–15 years, and 1 (5%) for 16–20 years.

### Are learners satisfied with the course?

Nineteen of the learners (95%) were satisfied to a great extent with the culminating assignment and 20 (100%) were satisfied to a great extent with the course topics, materials (e.g., syllabus, readings, videos), and the professor. They valued the inclusion of the patient narrative videos although some provided suggestions for improvement. As one learner summarizes:

I like the idea of the patient videos but felt that the individual clips were all so short and quick that it didn’t leave the impact I wanted…. I would have liked more active patient involvement (which I recognize isn’t practical for covid/online), or longer entries from the patients. It was a nice sentiment though.

Other learners expressed that the patient narrative videos, “enriched the course experience and content understanding” and that “the incorporation of the patient videos was phenomenal. I enjoyed the discussions had from them, and it helped to better understand the readings.”

### Following course completion, to what extent do learners perceive the course to be relevant to their professional practice?

Almost all learners found the course relevant to their professional practice to a moderate (n = 6; 30%) and great (n = 13; 65%) extent; only one (5%) found it relevant to a small extent. When asked about the extent to which each essential question (i.e., topic) was relevant to their professional practice, the results showed similar trends (see Table 1). However, most learners reported that involving patients in the development of admissions criteria and processes, as well as the development of educational curricula, was relevant only to a small (n = 7, 35%) or moderate (n = 7, 35%) extent.

### Following course completion, to what extent are learners confident in their abilities to actively involve patients in HPE?

The extent to which learners reported feeling confident in their abilities to actively involve patients in HPE was consistent across activities (see Table 2).

**Table 2:** Learners’ Perceived Confidence to Involve Patients in HPE.


TO WHAT EXTENT ARE LEARNERS CONFIDENT IN THEIR ABILITY TO ACTIVELY INVOLVED PATIENTS IN…	N	NOT AT ALL CONFIDENT	CONFIDENT TO A SMALL EXTENT	CONFIDENT TO A MODERATE EXTENT	CONFIDENT TO A GREAT EXTENT	I DON’T KNOW

		n (%)				

Development of educational experiences	20	0	1 (5)	13 (65)	5 (25)	1 (5)

Development of educational materials	20	0	2 (10)	10 (50)	7 (35)	1 (5)

Teaching learners	20	0	2 (10)	11 (55)	6 (30)	1 (5)

Mentoring learners	20	0	4 (20)	10 (50)	4 (20)	2 (10)

Assessment of learners	20	0	3 (15)	12 (60)	4 (20)	1 (5)

Provision of feedback to learners	20	0	2 (10)	11 (55)	6 (30)	1 (5)

HPE research	20	0	6 (30)	10 (50)	4 (20)	0


### How did learners perform on the culminating assignment?

The results indicated that learners attained course expectations. The culminating assignment was graded out of 35 points. The mean was 29.7 points (SD 2.9). The median was 29.9 (min 17.5, max 34.3).

## Critical Reflection

By providing learners with information on and strategies for actively involving patients in HPE and by collaborating with patients in the development of this course, we are hopeful that learners will integrate patients’ voices in their future educational practices. We also hope that many of the learners will become champions for active patient involvement in HPE. They can then teach their current and future colleagues and learners about the importance of active patient involvement in HPE as a means of creating a community of practice in this area and promoting compassionate healthcare. With this, we do not yet understand the contributions that this course has on learners’ practices as educators, mentors, or clinicians because the design does not include experiential learning that extends beyond the online course itself. We need to conduct longitudinal evaluations to explore if and how learners are applying their learning from the course in their practice. We now have a collection of innovations that the learners created as part of this course (i.e., culminating assignment) that we can draw on to expand the course evaluation. We will investigate if any learners have successfully implemented their innovations in their practices and what we can do to better support them in this implementation.

Noting that only half of the learners completed the post-course evaluation survey, we need to improve our efforts to engage learners in the ongoing evaluation of this course to ensure that the course is meeting all learners’ needs and achieving the intended learning outcomes. Continuing to draw on participatory and collaborative evaluation approaches, we can improve our stakeholder and participant engagement practices.

We are committed to understanding the experiences of the patients involved in the course and what we can do to improve our own patient involvement efforts [8]. We will also work with the advisory committee and the provincial patient advisory program to determine how we can better actively involve patients in the course itself. We were surprised that no learners requested to connect with the patients from the narrative videos. It is possible that this opportunity was viewed as an additional learning activity beyond the course. Thus, we need to increase the level of engagement between patients and learners in a way that is meaningful, sustainable for both parties, and not tokenistic within the context of this asynchronous, online course. Moreover, although patients who volunteered to participate in the narrative videos represented diverse age ranges, health conditions, and experiences in HPE, they were often Caucasian, educated, and from a higher socio-economic background [1,9]. Thus, they do not represent the broad diversity of patients that the learners will encounter and work with throughout their HPE careers [1]. A current problem in patient engagement, and one reflected in the development of this course, is the homogeneity of patients included in the course content. Increasing the diversity of patient representation in the course will help provide important insights and experiences to the learners [9].

While this course is unique, we recognize that it, designed as a graduate-level course embedded within our institution’s graduate HPE programs, limits accessibility to those enrolled in these programs. Remembering the broader sense in which we hope patient involvement practices to be deployed, we need to consider creative ways in which this course could be adapted, piloted, and evaluated within other learning contexts, such as integrating selected modules into other institutions’ existing HPE courses or workshops. Such efforts would facilitate the embedment of patient involvement into educational structures and priority areas of institutions [10] as well as demonstrate the important relationships between HPE and patient outcomes [11].
